# Differential expression of biomarkers in saliva related to SARS-CoV-2 infection in patients with mild, moderate and severe COVID-19

**DOI:** 10.1186/s12879-023-08573-6

**Published:** 2023-09-15

**Authors:** Lázaro Verdiguel-Fernández, Rene Arredondo-Hernández, Jesús Andrés Mejía-Estrada, Adolfo Ortiz, Antonio Verdugo-Rodríguez, Patricia Orduña, Samuel Ponce de León-Rosales, Juan José Calva, Yolanda López-Vidal

**Affiliations:** 1 Departamento de Microbiología Y Parasitología, Programa de Inmunología Molecular Microbiana, Facultad de Medicina, UNAM, CDMX, México; 2 Laboratorio de Microbioma, División de Investigación, Facultad de Medicina, UNAM, CDMX, México; 3 Departamento de Microbiología E Inmunología, Laboratorio de Microbiología Molecular, Facultad de Medicina Veterinaria Y Zootecnia, Universidad Nacional Autónoma de México, CDMX, México; 4 Departamento de Microbiología E Inmunología, Unidad de Bioseguridad de Brucella, Facultad de Medicina Veterinaria Y Zootecnia, Universidad Nacional Autónoma de México, CDMX, México; 5 Programa Universitario de Investigación en Salud, Universidad Nacional Autónoma de México, CDMX, México; 6 Departamento de Infectología, Instituto Nacional de Ciencias Médicas y Nutrición “Salvador Zubirán”, CDMX, México

**Keywords:** COVID-19, Biomarkers, Cytokines, ACE2, IDO1, Saliva, Severity

## Abstract

**Background:**

Severe COVID-19 is a disease characterized by profound dysregulation of the innate immune system. There is a need to identify highly reliable prognostic biomarkers that can be rapidly assessed in body fluids for early identification of patients at higher risk for hospitalization and/or death. This study aimed to assess whether differential gene expression of immune response molecules and cellular enzymes, detected in saliva samples of COVID-19 patients, occurs according to disease severity staging.

**Methods:**

In this cross-sectional study, subjects with a COVID-19 diagnosis were classified as having mild, moderate, or severe disease based on clinical features. Transcripts of genes encoding 6 biomarkers, IL-1β, IL-6, IL-10, C-reactive protein, IDO1 and ACE2, were measured by RT‒qPCR in saliva samples of patients and COVID-19-free individuals.

**Results:**

The gene expression levels of all 6 biomarkers in saliva were significantly increased in severe disease patients compared to mild/moderate disease patients and healthy controls. A significant strong inverse relationship between oxemia and the level of expression of the 6 biomarkers (Spearman’s correlation coefficient between -0.692 and -0.757; *p* < 0.001) was found.

**Conclusions:**

Biomarker gene expression determined in saliva samples still needs to be validated as a potentially valuable predictor of severe clinical outcomes early at the onset of COVID-19 symptoms.

**Supplementary Information:**

The online version contains supplementary material available at 10.1186/s12879-023-08573-6.

## Background

Coronavirus disease 2019 (COVID-19), caused by severe acute respiratory syndrome coronavirus 2 (SARS-CoV-2), has led to an ongoing global pandemic, gravely affecting public health and causing millions of deaths worldwide [1–3]. The clinical manifestations and prognosis of COVID-19 are highly variable, and although the great majority of patients show a mild and benign clinical presentation, a significant proportion of infected subjects quickly develop severe pulmonary symptoms, including acute respiratory distress syndrome, multiple organ failure and death, after illness onset. Dysregulation of the host immune response with activation of inflammatory cytokines and coagulopathy has been associated with disease severity and poor prognosis [4, 5].

Certain comorbidities (such as hypertension, diabetes, obesity, cardiopulmonary diseases, immunosuppression and asthma) and other conditions (such as smoking and advanced age) have been firmly established as risk factors for disease severity and mortality [6, 7]. Some risk stratification tools that predict in-hospital mortality or in-hospital clinical deterioration (defined as requiring ventilatory support or critical care) in hospitalized COVID-19 patients have been developed [8]. Yet, prognostic scales using biomarkers have been less developed in patients seen in out-patient clinics with non-severe illness, at the onset of the disease. Previous reports indicate that changes in some biomarkers, such as certain cytokines and other inflammatory mediators, can be used to assess the severity of COVID-19. To our knowledge, the majority of these reports come from the study of blood samples and nasopharyngeal and bronchoalveolar swabs and there is a need to stratify patients according to disease severity using non-invasive samples such as saliva. This will facilitate the early identification of individuals who need timely interventions (such as preemptive antiviral therapy, close health monitoring, etc.) to prevent catastrophic clinical outcome [9].”

Several molecules involved in the pathogenesis of severe COVID-19 have been identified and quantified in blood samples of SARS-CoV-2 infected individuals. In particular, certain proinflammatory biomarkers, such as interleukin (IL)-1β, IL-6, IL-10 and C-reactive protein (CRP), and cellular enzymes, such as indoleamine-2,3-dioxygenase 1 (IDO1) and angiotensin-converting enzyme 2 (ACE2), have been found to be increased in the sera of patients with severe illness compared with subjects with milder forms of the disease. Furthermore, a longitudinal study reported that circulating ACE2 in plasma could be used to predict the outcome of COVID-19 in hospitalized patients [10–14].

Host transcriptome studies in patients with COVID-19 have revealed distinct host inflammatory cytokine gene expression profiles. In a recent study, RNA sequencing of nasopharyngeal fluid swab samples was performed among patients with mild, moderate or severe illness, and a molecular signature associated with disease severity was found [15].

Meta-analyses based on studies of clinical laboratory findings comprise an additional tool for predicting the severity of COVID-19 and have revealed that the cytokine storm represents one of the main determinants of the progression and deterioration of pneumonia related to SARS-CoV-2 and that lymphopenia, thrombocytopenia, and elevated levels of IL-6, ferritin, D-dimer, aspartate aminotransferase, CRP, procalcitonin, creatinine, neutrophils, and leukocytes are associated with severe disease and death from COVID-19 [16, 17]. In addition, several studies have reported a correlation between the upregulation of IL-6 and elevated plasma levels of CRP, IL-10, and IL-1β as well as an increase in the expression levels of ACE2 and IDO1 [18–22].

Whether a similar association with disease severity is found when transcripts of such molecules are measured in saliva samples is of interest because these samples are easier and safer to obtain and potentially more useful in routine clinical practice for assessing markers for early identification of patients at increased risk for developing severe illness.

As a first step to accomplish this goal, we tested, through a cross-sectional study, whether gene expression in saliva samples of the above mentioned proinflammatory molecules and cellular enzymes is increased in COVID-19 patients with clinical signs of severe lung damage. These data could become the rationale for assessing whether such biomarkers could serve as accurate baseline predictors of progression to severe illness at early stages of the disease in future cohort studies.

## Methods

### Study design

This was an observational cross-sectional survey of 2 study samples: one group of subjects with mild, moderate, and severe COVID-19 and a control group of COVID-19-free individuals.

### Study population

Eligible participants aged between 17 and 67 years of both sexes were selected from a population of subjects with signs and symptoms suggestive of possible COVID-19, i.e., having at least two of the following symptoms in the last 7 days: fever, cough, headache, myalgia, chest pain, dyspnoea, and/or a disturbance in taste and/or olfaction. The participants had visited a university outpatient health care facility (UNAM, in Mexico City) for medical care, and a SARS-CoV-2 RT‒qPCR test was carried out on saliva samples from each patient between January and March 2021. A positive RT‒qPCR test defined a case of confirmed COVID-19. Asymptomatic volunteers with a negative SARS-CoV-2 RT‒qPCR test result in saliva were included as negative controls. All participants provided an informed consent letter for sample collection and subsequent analysis. No subject with asymptomatic SARS-CoV-2 infection was included. Patients with critical illness (acute respiratory distress syndrome) are usually referred to hospitals; these patients were not included in this study.

### Clinical assessment

In all participants with confirmed COVID-19, heart rate, respiratory rate, blood pressure, body temperature and oxygen saturation index as measured by pulse oximetry (SpO_2_) were recorded, and a smell test was performed to assess the severity of olfactory impairment. Subjects were asked about specific clinical features, including 12 different symptoms (namely, headache, rhinorrhoea, conjunctivitis, cyanosis, polypnea, chills, abdominal pain, diarrhoea, vomiting, chest pain, loss of appetite and seizures) and related risk factors such as diabetes, hypertension, obesity, chronic obstructive pulmonary disease (COPD), asthma, heart disease, chronic kidney failure, immunosuppression and smoking.

### COVID-19 Disease severity classification

Cases were classified as mild, moderate or severe illness according to the World Health Organization (WHO) criteria [3]. Mild disease was defined as symptomatic patients meeting the case definition for COVID-19 without clinical evidence of viral pneumonia (i.e., the absence of fever, cough, dyspnoea and fast breathing) or hypoxia (SpO2 =  > 90% on room air); moderate disease as those with at least one clinical sign of pneumonia (fever, cough, dyspnoea and/or fast breathing), without hypoxia (SpO2 =  > 90% on room air); and severe disease as those with at least one clinical sign of pneumonia plus at least one of the following: respiratory rate > 30 breaths/min or SpO2 < 90% on room air.

This study followed the Declaration of Helsinki ethical principles, and the research protocol was approved by the Facultad de Medicina-UNAM Institutional Ethics Committee (FM/DI/047/2020).

### Saliva sample collection and RNA extraction

Saliva samples were collected following medical consultation and prescription and signature of the informed consent form letter. Two millilitres of saliva were collected and mixed with the same volume of viral transport medium (Hank’s solution with added antibiotics) [23]. Samples were kept at 4 °C, and RNA was extracted within the next four hours.

In saliva samples, total RNA was extracted and quantified using the TRIzol assay (Sigma, Life Science, St. Louis, USA). Viral RNA was obtained using the QIAamp Viral RNA Mini Kit technique (QIAGEN®, Germany) following the manufacturer’s instructions.

### Molecular detection of SARS-CoV-2 by RT‒qPCR in saliva

SARS-CoV-2 genome detection was performed by using the COVID-19 Plus Real*AMP* Kit (GeneFinder™ REF: IFMR-45, South Korea) targeting the viral E, N, and RdRp genes, with the RNase P gene as an extraction control. Amplification was carried out in a 7500 real-time detection system and analysed with 7500 software v2.3 (Applied Biosystems, Massachusetts, USA) following the manufacturer’s instructions. Briefly, thermocycling conditions were as follows: a single cycle of 20 min at 50 °C and 5 min at 95 °C, followed by 45 cycles of 95 °C for 15 s and 58 °C for 30 s. An amplification signal below a threshold cycle of 37, plus RNAase P CT below 25, was considered reliable.

### Measurement of the relative expression of biomarkers in saliva

Saliva samples from COVID-19 patients (classified as having mild, moderate, and severe disease) and from COVID-19-free subjects were assessed. To quantify the relative expression of the biomarker genes, a real-time RT‒qPCR assay was performed using GoTaq® Probe Real-Time One-Step RT‒PCR Master Mix (Promega, Wisconsin, USA). A total reaction volume of ​​20 μl was used, containing 10 μl of 1X GoTaq® Probe qPCR Master Mix with dUTP, 0.4 μl of GoScript™ RT Mix for 1-Step RT‒qPCR, 1 μl of each forward and reverse oligonucleotide primer to obtain a final concentration of 500 nM, 2.1 μl of nuclease-free water, 0.5 μl of each probe for a final concentration of 250 nM, and 5 μl of RNA extracted from the clinical samples. Amplification was carried out in 96-well plates using a 7500 real-time detection system and 7500 software v2.3 (both from Applied Biosystems, Massachusetts, USA). The thermocycling conditions were as follows: 15 min at 45 °C for reverse transcription, 2 min at 95 °C for the activation of the AmpliTaq Gold DNA polymerase, and 45 cycles of 15 s at 95 °C and 30 s at 60 °C [24]. The obtained data were normalized using the housekeeping gene hypoxanthine phosphoribosyl transferase 1 (HPRT1; TaqMan**™** Assay Human HPRT1, Applied Biosystems, Massachusetts, USA) as the reference gene. To determine the relative mRNA expression levels, the double delta Ct method (ΔΔCt) was used, which expresses the ratio obtained from the relationship between the Ct values ​​of the sample and those ​​of the constitutive control as shown in the following equation:  [25].

$$Ratio\;=2-\left(\Delta Ct\;sample-\Delta Ct\;reference\right)\Rightarrow Ratio=2-\Delta\Delta Ct$$

For the amplification of the IL-6 (GenBank: M54894.1), IL1β (GenBank: M15330.1), and CRP (GenBank: M11880.1) genes, primers were designed based on the sequences reported in the NCBI. Bioinformatic analysis was performed to verify the alignment temperature and that the primers did not form dimers or hairpin loops. BLAST analysis was performed to verify the specificity of the primers; for the three genes, 100% identity with the reported sequences was obtained. To perform these bioinformatic analyses, Snapgene Viewer (from Insightful Science; available at snapgene.com) and Vector NTI (software by BioScience Technology) software were used. For the amplification of the IL-10, IDO1 and ACE2 genes [24, 26, 27], primers reported by other authors were used. The primer and probe sequences we used are listed in Table 1.

**Table 1:** List of primers and probes used to amplify the genes for 3 cytokines, C-reactive protein and 2 cellular enzymes

Gene	Primer	Sequence	Size fragment (bp)	Reference
**IL-1β**	**Forward** **Reverse** **Probe**	TACGAATCTCCGACCACCACT GGTGCTCAGGTCATTCTCCTG CGTCAGTTGTTGTGGCCATGGA (FAM)	**128 bp**	This work
**IL-6**	**Forward** **Reverse** **Probe**	TGACCCAACCACAAATGCCAG AGGTGCCCATGCTACATTTGC TGCAGGCACAGAACCAGTG (FAM)	**150 bp**	This work
**IL-10**	**Forward** **Reverse** **Probe**	GTGATGCCCCAAGCTGAGA CACGGCCTTGCTCTTGTTTT CCAAGACCCAGACATCAAGGCGCA (FAM)	**138 bp**	[16]
**CRP**	**Forward** **Reverse** **Probe**	AAGCCTTCACTGTGTGCCTC GGAACTGTCCTCGACCCGTGGGT CAGACCCACCCACTGTAAAACT (FAM)	**150 bp**	This work
**IDO1**	**Forward** **Reverse** **Probe**	CTGGGCATCCAGCAGACT TGAGCTGGTGGCATATATCTTCT GAGGACATGCTGCTCAGTT (FAM)	**100 bp**	[18]
**ACE2**	**Forward** **Reverse** **Probe**	TCCATTGGTCTTCTGTCACCCG AGACCATCCACCTCCACTTCTC CCTGCTCAAACAAGCACTCACG (FAM)	**133 bp**	[19]

### Statistical analysis

To compare demographic and clinical features among the 4 groups (mild, moderate, and severe COVID-19 cases and COVID-19-free subjects), significant differences in categorical data were analysed with the NxK chi-square test, and for continuous data, the nonparametric Kruskal‒Wallis one-way ANOVA by ranks test was used; *p* values less than 0.05 were considered statistically significant.

To compare the level of expression of biomarkers in saliva (numerical variable) among the 4 groups (mild, moderate, and severe COVID-19 cases and the COVID-19-free subjects), the nonparametric Kruskal‒Wallis one-way ANOVA by ranks test was used. Pot hoc pairwise comparisons were made by using Dunn’s multiple comparisons procedure; *p* values less than 0.05 were considered statistically significant.

The magnitude of the association between the level of expression of the 6 biomarkers in saliva (among themselves) and the oxygen saturation index was classified according to the value of the nonparametric Spearman´s rho correlation coefficient: 0–0.19 = very weak correlation; 0.2–0.39 = weak correlation; 0.4–0.59 = moderate correlation; 0.6–0.79 = strong correlation; and 0.8–1 = very strong correlation. Statistical analysis was performed using GraphPad Prism 8.0 (GraphPad Software, USA).

Principal component analysis (PCA) was carried out to further assess the relationship between the expression levels of the 6 biomarkers and disease severity clustering. PCA was performed by using the Microsoft Excel 365 plugin XLSTAT (Addinsoft, New York, USA). A Promax rotation was used, and biplots were plotted.

A classification and regression tree analysis (CART) was performed among parametrized variables to determine the best biomarker range, combination, and performance in severity segregation. The clinical manifestation of the disease (mild, moderate, or severe) was set as the dependent outcome of the previously quantified cytokines and biomarkers, whereas a CHAID algorithm was used with a tree branch depth of 3; the significance level was set at 5%, and a verification step was performed using one random case, which was correctly categorized.

### Data availability statement

The datasets generated during the current study are available from the corresponding author on reasonable request.

## Results

**Fig. 1:**
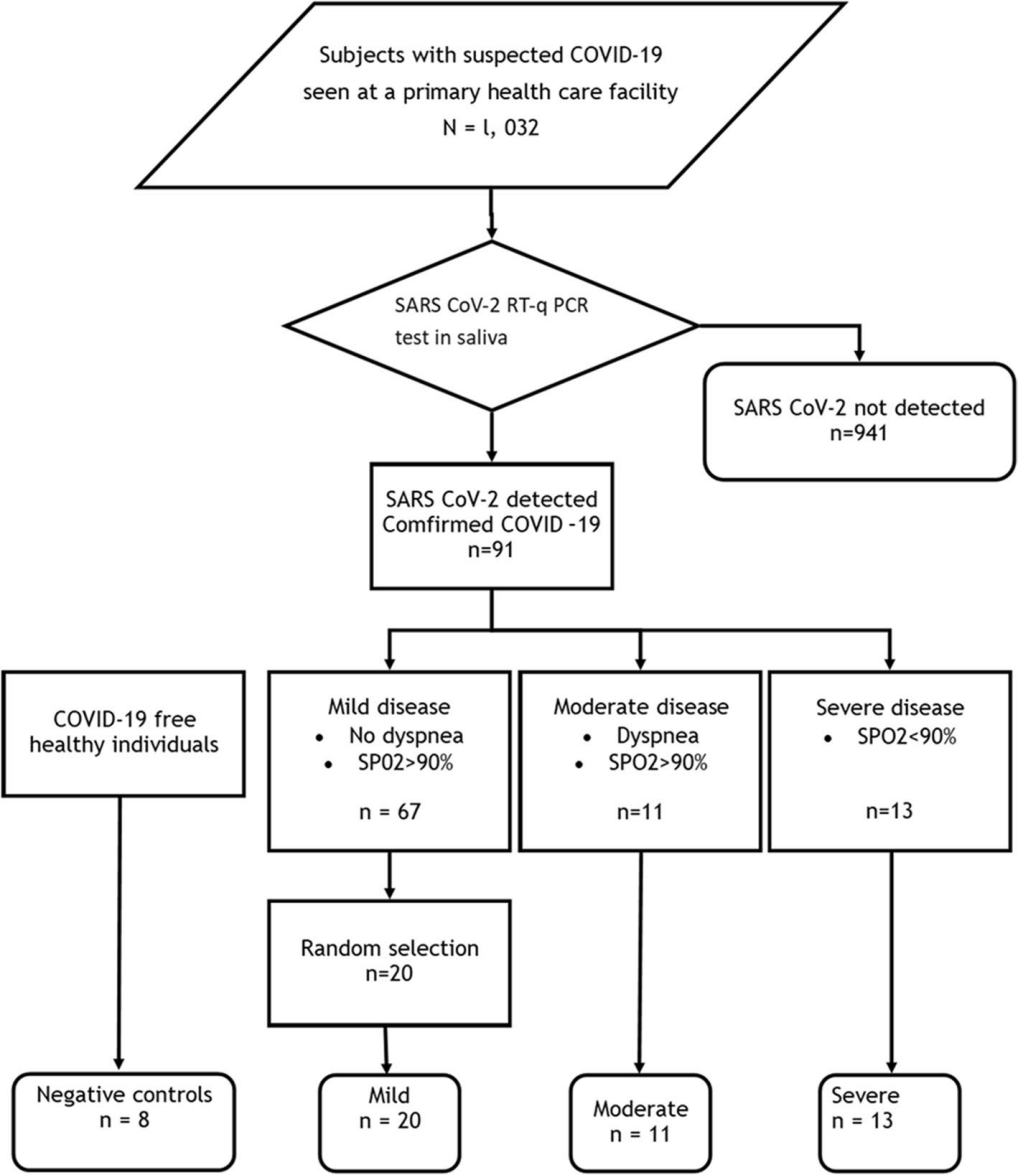
Study profile. Selection of 52 study individuals

Ninety-one subjects (9%) were identified as patients with confirmed COVID-19 from 1,032 individuals with suspected COVID-19 seen at the university primary health care facility. Of the 91 subjects, 66 presented mild disease (20 of them were randomly selected), 11 moderate disease and 13 severe diseases. These 44 patients with COVID-19, in addition to 8 COVID-19-free volunteer participants (52 participants in total), were included in the study measuring the level of biomarker expression in saliva (Fig. 1). Two selected patients with mild disease and one with moderate disease had long COVID-19 (i.e. subjects with symptoms lasting more than 8 weeks at the time of saliva sampling). None of the subjects included in this study had been immunized against COVID-19. Saliva sampling was carried out between January and March 2021 and, in Mexico, vaccination against COVID-19 for the general population began in June 2021.

Table 2 shows the comparison of participants’ demographic and clinical features among the 3 disease severity categories and the control group. Compared to individuals with mild disease or no disease (control group), subjects with moderate and severe disease were older and more frequently had dyspnoea, chest pain, tachycardia, and hypoxemia.

**Table 2:** Demographic and clinical features of 44 patients with COVID-19, according to disease severity staging, and 8 COVID-19-free (control) individuals

Features		COVID-19 severity stage				
	Control 8 individuals n (%)	Mild 20 cases n (%)	Moderate 11 cases n (%)	Severe 13 cases n (%)	Total 52 participants n (%)	*p* value*
Men	4 (50)	9 (45)	5 (45)	8 (61.5)	26 (50)	NS
Women	4 (50)	11 (55)	6 (55)	5 (38.5)	26 (50)	NS
Age in years: median (p25-p75)	30 (26–30.7)	36 (23–48.7)	37 (27–48)	48 (36–54.5)	37 (25.5–48.7)	NS
Fever	0 (0)	3 (15)	2(18.8)	6 (46.2)	11 (21.2)	0.025
Headache	0(0)	12 (60)	6 (54.5)	9 (69.2)	27 (51.9)	0.013
Cough	0(0)	8 (40)	7(63.6)	11 (84.6)	26 (50)	0.001
Dyspnoea	0(0)	0 (0)	11 (100)	6 (46.1)	17(32.7)	< 0.001
Chest pain	0(0)	4 (20)	7 (63)	10 (76.9)	21 (40.3)	0.001
Olfaction disorder	0(0)	8 (40)	5 (45.5)	5 (38.5)	18 (36.6)	NS
Myalgias	0(0)	8 (40)	5 (45.5)	8 (61.5)	21 (40.4)	0.047
Heart rate: median (p25—p75) beats per minute	69 (65.7–71.5)	86 (78–88.7)	80 (75–92)	93 (84–111.5)	85 (73.5–90)	NS
Oxygen saturation index: % (median 95%-CI)	96 (95.1–96.8)	94 (93–95.8)	93 (92–95)	89 (87.5–89)	93 (89.3–95)	< 0.001
Diabetes	0(0)	3 (15)	1 (9.1)	1 (7.7)	5 (9.6)	NS
Hypertension	0(0)	2 (10)	1 (9.1)	3 (23.1)	6 (11.5)	NS
Obesity	0(0)	7 (35)	4 (36.3)	8 (61.5)	19 (36.5)	0.043
Smoking	0(0)	2 (10)	0 (0)	2 (15.4)	4(7.7)	NS

*NS* Nonsignificant

^*^to assess differences among the 4 subgroups, categorical variables were analysed with a 2 × 4 chi^2^ test and quantitative variables with the Kruskal‒Wallis test

### Differential biomarker gene expression in saliva according to disease severity in 44 COVID-19 patients and in 8 disease-free individuals

**Fig. 2:**
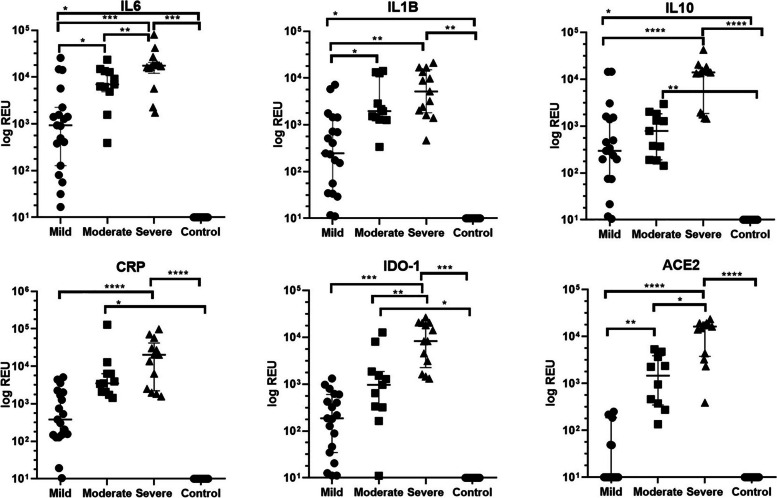
Distribution of the relative expression units (REU) of the 6 biomarkers according to the 4 study groups: 20 patients with mild COVID-19, 11 with moderate COVID-19 and 13 with severe COVID-19 and the control group (8 COVID-19-free individuals). IL = interleukin, CRP = C-reactive protein, IDO1 = indoleamine-2,3-dioxygenase 1, ACE2 = angiotensin-converting enzyme 2. Horizontal bars indicate the median value. Asterisks indicate the *p* value for the pairwise comparisons: * *p* =  < 0.05, ** *p* =  < 0.01, *** *p* =  < 0.001 and **** *p* =  < 0.0001

Figure 2 shows that the distribution of the relative expression units of the 6 studied biomarkers in saliva was different among subgroups of COVID-19 patients according to disease presentation (mild, moderate and severe).

The expression levels of IL-6, IL-1β, IL-10, CRP, IDO1 and ACE2 were significantly higher in subjects classified as having severe illness than in those with mild or moderate illness and disease-free subjects. Patients with mild/moderate illness showed significantly higher expression levels of IL-6, IL-1β, IL-10, CRP and IDO1 than COVID-19-free subjects.

**Fig. 3:**
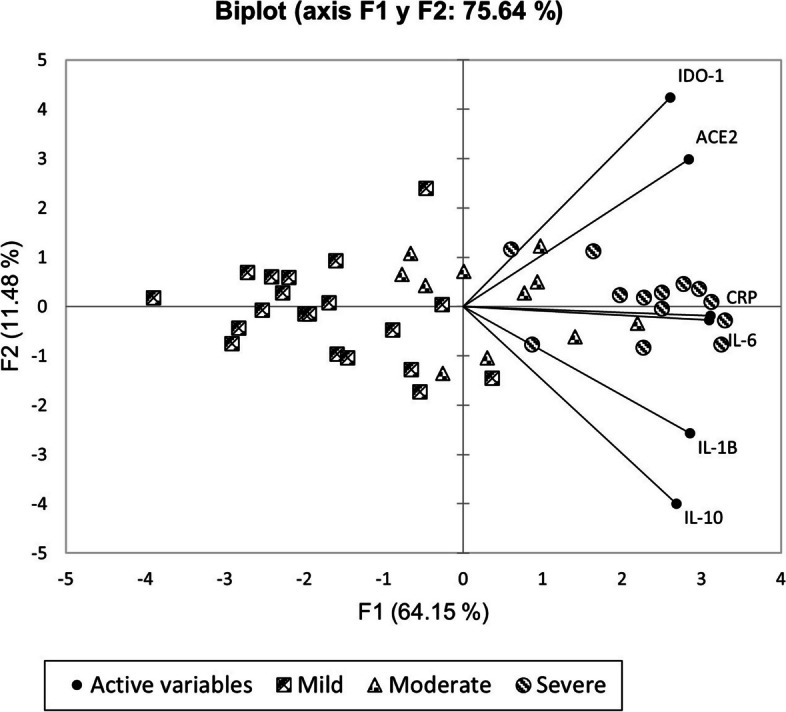
Biplot principal component analysis (F1 and F2) of the relative biomarkers expression in saliva and their association with COVID-19 severity classification strata. IL = interleukin, CRP = C-reactive protein, IDO1 = indoleamine-2,3-dioxygenase 1, ACE2 = angiotensin-converting enzyme 2

Table 3 and Fig. 3 illustrate the PCA results. The biplot cluster of subjects according to disease severity showed that the most influential variables (as component F1) were the expression levels of IL-6, IL-1β, IL-10 and CRP, which explained 64.15% of the data variance, whereas the next most influential variables (component F2) were the expression levels of ACE2 and IDO1, which explained 11.48% of the data variance (75.64% of the cumulative variance).

**Table 3:** Eigenvalues of variables for each vector after Promax rotation

	D1	D2
IL-6	**0.415**	0.257
IL-1β	**0.672**	0.025
IL-10	**0.787**	0.002
CRP	**0.407**	0.275
IDO-1	0.000	**0.779**
ACE2	0.032	**0.688**

The values ​​in bold for each variable correspond to the factor for which the cosine squared is higher

**Fig. 4:**
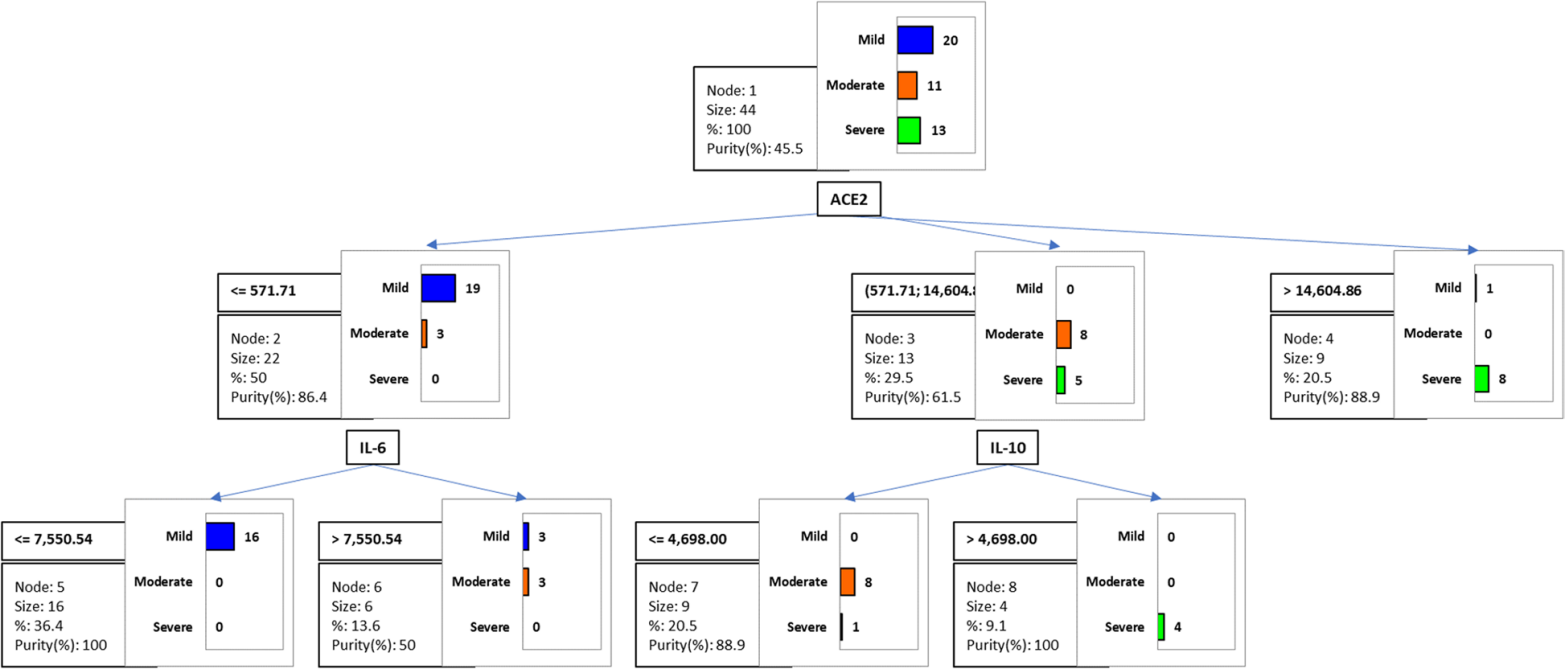
Classification and regression tree. Node 1: root node. Node 2: if ACE2 expression is ≤ 571.71, then 50% are mild cases. Node 3: if ACE2 expression is between 331.5 and 571.71, then 9.8% are moderate cases. Node 4: if ACE2 expression is between 571.71 and 14604.86, then 31.7% are moderate cases. Node 5: if ACE2 expression is > 14604.86, then 19.5% are severe cases. Node 6: if ACE2 expression is between 571.71 and 14604.86 and IL-10 expression is ≤ 4698, then 22% are moderate cases. Node 7: if ACE2 expression is between 571.71 and 14604.86 and IL-10 expression is > 4698, then 9.8% are severe cases

A regression tree analysis showed the power of the expression of ACE2, IL-6 and IL-10 to differentiate between the 3 strata of disease severity (Fig. 4). A level of ACE2 expression equal to or less than 571.71 relative expression units (REU) corresponded to mild disease (node 2), whereas a value greater than 14,604.86 REU corresponded to severe illness (node 4). If this biomarker was expressed at less than 571.71 REU, a level of expression of IL-6 less than or greater than 7550.4 REU distinguished mild and moderate disease, respectively (nodes 5 and 6). If an intermediate level of the ACE2 biomarker (between 571.71 and 14,604.86 REU) was observed, a level of expression of IL-10 equal to or less than 4,698 REU corresponded to mild/moderate disease (node 7), whereas a value greater than this number was associated with severe disease (node 8). When applying this algorithm, patients were correctly classified as having mild (in all cases), moderate (in 80% of cases) and severe disease (in 90% of cases).

### Interrelationship of the level of expression among the 6 biomarkers and with the level of oxemia and age

Supplementary Fig. 1 and Table 4 included the variables that were used to classify the clusters according to disease severity taking into account the expression levels of IL-1β, IL-6, IL-10, CRP, IDO1, and ACE2 (biomarkers). In this CART analysis, a regression tree with a total of 6 nodes was obtained. The root node was divided into two nodes (2, 3) according to the SpO_2_, distinguishing severe disease with 100% purity (node ​​2) and moderate and mild disease with 64.5% purity (node ​​3). Node 3 was divided into three nodes according to ACE2 expression levels, distinguishing subjects with mild disease with a purity of 100% (node ​​4), subjects with mild and moderate disease with a purity of 66.7% (node ​​5), and subjects with moderate and mild disease with a purity of 88.9% (node 6). The CART decision rules are explained in detail in Supplementary Fig. 1.

**Table 4:** Spearman’s rho correlation coefficient matrix as a measure of the relationship between interleukin (IL)-1β, IL-6, IL-10, C-reactive protein (CRP), indoleamine-2,3-dioxygenase 1 (IDO1), angiotensin-converting enzyme 2 (ACE2), age and oxygen saturation index (SpO_**2**_) in 44 study subjects with COVID-19

Variable	Age	SpO_2_	IL-6	IL-1 β	IL-10	CRP	IDO	ACE2
Age	1.000							
SpO_2_	-.526^**^	1.000						
IL-6	0.386^**^	-0.757^**^	1.000					
IL1-β	0.321^*^	-0.726^**^	0.782^**^	1.000				
IL-10	0.284^*^	-0.720^**^	0.782^**^	0.745^**^	1.000			
CRP	0.240	-0.692^**^	0.840^**^	0.811^**^	0.755^**^	1.000		
IDO	0.328^*^	-0.741^**^	0.725^**^	0.735^**^	0.663^**^	0.769^**^	1.000	
ACE2	0.264	-0.713^**^	0.737^**^	0.661^**^	0.611^**^	0.757^**^	0.672^**^	1.000

*p* values: * *p* < 0.05 ** *p* < 0.01

Table 4 shows the correlation coefficient matrix between all these variables. The following associations were found: a strong positive relationship of the level of expression of all 6 biomarkers among themselves, a strong inverse relationship between the level of expression of the 4 inflammatory biomarkers and of the 2 cellular enzymes with the oxygen saturation index and a weak positive association between the level of expression of all 6 biomarkers and age.

## Discussion

Based on our knowledge of the natural history of COVID-19, a significant proportion of diseased subjects (both with and without risk factors for severity) present sudden clinical deterioration (eventually leading to hospitalization and death) after approximately one week with mild signs and symptoms after illness onset. It has been postulated that these adverse outcomes are secondary to an immune hyperreactivity phenomenon (“cytokine storm”), while it is very difficult to predict, on clinical grounds alone during the first days of illness, who will (or will not) eventually suffer such immune dysfunction with organ failure [28, 29]. The latter constitutes the rationale for the search for reliable biomarkers as early predictors of immune dysfunction that antedate the surge in adverse clinical events; this would allow the timely identification of those patients requiring and benefiting from a closer surveillance of vital signs and by the use of preemptive efficacious antiviral or immunomodulatory agents [12, 30, 31].

Diverse host molecules have been widely identified as surrogate markers of viral activity and of immune hyperactivity and hypercoagulability. Diverse innate immune response molecules, such as serum C-reactive protein, erythrocyte sedimentation rate, ferritin, procalcitonin, amyloid A, IL-1β, IL-2, IL-2R, IL-4, IL-6, IL-8, IL-10, tumour necrosis factor-alpha (TNFα), interferon-gamma (IFNγ) and D-dimer, have been measured in COVID-19 patients. Several clinical studies have consistently documented a relationship between the levels of these molecules in sera and the illness-severity strata [19–21, 32–36]. These observations are in accordance with the postulated pathogenesis of deleterious COVID-19 clinical outcomes [9, 13]. However, given their cross-sectional nature, the majority of these studies do not provide data on how many days the increased concentration of these molecules antedated organ failure [16, 17, 37, 38].

Few of these studies are longitudinal (cohort) studies showing that blood concentrations of C-reactive protein and ACE2 in basal samples obtained at hospital admission are increased in patients presenting with subsequent clinically overt organ deterioration [31–34]. These data suggest their potential useful role as predictors of disease severity, pending the estimation of their actual positive and negative predictive values as indices of test diagnostic performance [19, 33, 39–42].

Interestingly, other cross-sectional studies have assessed the transcriptional signatures of the host inflammatory response to SARS-CoV-2 infection through transcriptome sequencing of RNAs isolated from peripheral blood mononuclear cells, serum, nasopharyngeal exudate and bronchoalveolar lavage fluid specimens obtained from COVID-19 patients. These studies have revealed distinct inflammatory cytokine profiles according to disease severity [15, 32].

We were interested in investigating whether the gene expression levels of some of these biomarkers could be measured in saliva, which constitutes an easier to obtain body fluid with less discomfort to the patient and with less risk of viral contagion to health workers. Our data show that the expression levels of IL-6, IL-1β, IL-10, CRP, IDO1 and ACE2 were significantly higher in subjects classified as having severe illness than in those with mild or moderate illness and that patients with mild/moderate illness had significantly higher expression levels of IL-6, IL-1β, IL-10, CRP and ACE2 than COVID-19-free (control) subjects. In our study, severity was defined based on signs of respiratory failure; accordingly, in most of the patients classified as having severe illness, the allocation was because they presented hypoxemia (i.e., an oxygen saturation index less than 90%). Differential expression of the 6 biomarkers in severe cases is further supported by the correlation analysis showing a significant strong inverse relationship between the level of expression of the biomarkers and the value of the oxygen saturation index. Moreover, for the majority of the 6 biomarkers, we found a moderate positive relationship in their expression among themselves, suggesting that in an individual, there is a trend in the same direction and magnitude for most of the biomarkers.

We carried out principal component analysis to further explore the relationship between the levels of the 6 biomarkers in saliva and patient clustering according to the clinical presentation of COVID-19. The expression levels of IL-6, IL-10 and ACE2 yielded high-magnitude vectors, indicating a significant role of these biomarkers in discriminating between the 3 disease groups. This finding fits with the observed clear differences in the distribution of the relative expression units of these 3 molecules between the 3 severity strata.

Similar results have been documented in other studies. Higher serum concentrations of ACE2 have been associated with obesity, hypertension and lung cancer (considered risk factors for severe disease) [39, 43], and in other studies, a direct correlation of ACE2 with severity and mortality has been found, independent of such comorbidities [44]. Moreover, increased serum levels of IL-10 have been observed in patients with critical COVID-19 compared with patients with severe or moderate illness [13, 42, 45].

Other studies have reported that most severe cases and deaths from COVID-19 are associated with dysregulation of the immune system because SARS-CoV-2 infection is accompanied by an exacerbated inflammatory response resulting from the release of proinflammatory cytokines and chemokines by immune effector cells, known as a cytokine storm [12]. In this context, our results show a statistically significant increase in the expression levels of IL-6, CRP, IDO1 and ACE2 in patients with severe disease compared to patients with mild and moderate COVID-19. In the case of IL-1β and IL-10, statistically significant differences were detected between the group with severe disease and that with mild disease and no disease. Additionally, studies of transcriptomic expression profiles in patients with COVID-19 in samples of mononuclear peripheral blood cells, bronchoalveolar lavage fluid and nasopharyngeal swabs show increased expression levels of IL-6 and other cytokines, such as IL-10, IL-8, CXCL10/IP-10, CCL2/MCP-1 and CCL3/MIP-1A, as well as an increase in the expression levels of ACE2 and CRP [32]. These studies´ results, showing a differential gene expression, have been confirmed by several other investigations reporting that patients with severe COVID-19 show elevated plasma levels of IL2, IL6, IL7, IL10, GSCF, IP10, MCP1, MIP1A, IL1β and TNFα compared to those with mild COVID-19, indicating that the release of inflammatory cytokines is critical in the progression of COVID-19 [31, 37]. These findings were compared with the results obtained in this study, revealing that IL-6 is an major indicator that can be used to predict the course of COVID-19 [33].

Regarding biomarkers evaluated in long COVID-19 patients, other studies have reported that these patients exhibited higher levels of proinflammatory cytokines/chemokines [IL-6, tumour necrosis factor alpha (TNF-), IL-1α, IL-1β, IFN γ, IL-17, IL-10, and C–C motif chemokine ligand (CCL) 2] and acute phase proteins [C-reactive protein (CRP) and ferritin] [46–48]. We detected an increase in the relative expression of the IL-6, CRP, ACE2 and IL-1β biomarkers of 2 mild cases and one moderate case with long COVID-19 compared to healthy individuals (Fig. 2).

In this cross-sectional study, we show that gene transcripts of certain cytokines, C-reactive protein, and some cellular enzymes, all involved in the pathogenesis of severe COVID-19, can be measured in the saliva of individuals with the disease. Furthermore, we documented the differential expression of these molecules, as patients with severe illness (clinically defined by respiratory failure) showed a significantly higher concentration of biomarker transcripts in saliva than symptomatic subjects without hypoxemia or increased breath rate.

A limitation of our investigation is the cross-sectional study design which does not allow the assessment of the prognostic accuracy of baseline biomarkers in the prediction of disease outcome.

Future studies aimed at assessing whether overexpression of these inflammatory markers and cellular enzymes in saliva can be detected days before respiratory (or multiorgan) failure need to be carried out through well-designed cohort studies. Hence, measurement (at early stages of COVID-19 disease) of biomarker transcripts in saliva samples could constitute a powerful approach to quantifying host molecular responses and an accurate and more convenient method to timely identify individuals at higher risk of immune-mediated severe organ damage and unfavourable clinical outcomes. Furthermore, follow-up of biomarkers over the progression of the illness may provide further understanding of COVID-19 pathogenesis.

Finally, it is desirable to evaluate more biomarkers; consequently, one of the directions of future work will be to analyse their transcriptome in saliva samples.

### Supplementary Information

**Additional file 1: Supplementary Figure 1.**

## Data Availability

Datasets used during the current study are available and can be requested from the corresponding author.
