# Supplementary figures and images for: Differential expression of biomarkers in saliva related to SARS-CoV-2 infection in patients with mild, moderate and severe COVID-19
Source: BMC Infect Dis. 2023 Sep 15;23:602. doi: 10.1186/s12879-023-08573-6 (PMC10502992; doi:10.1186/s12879-023-08573-6)

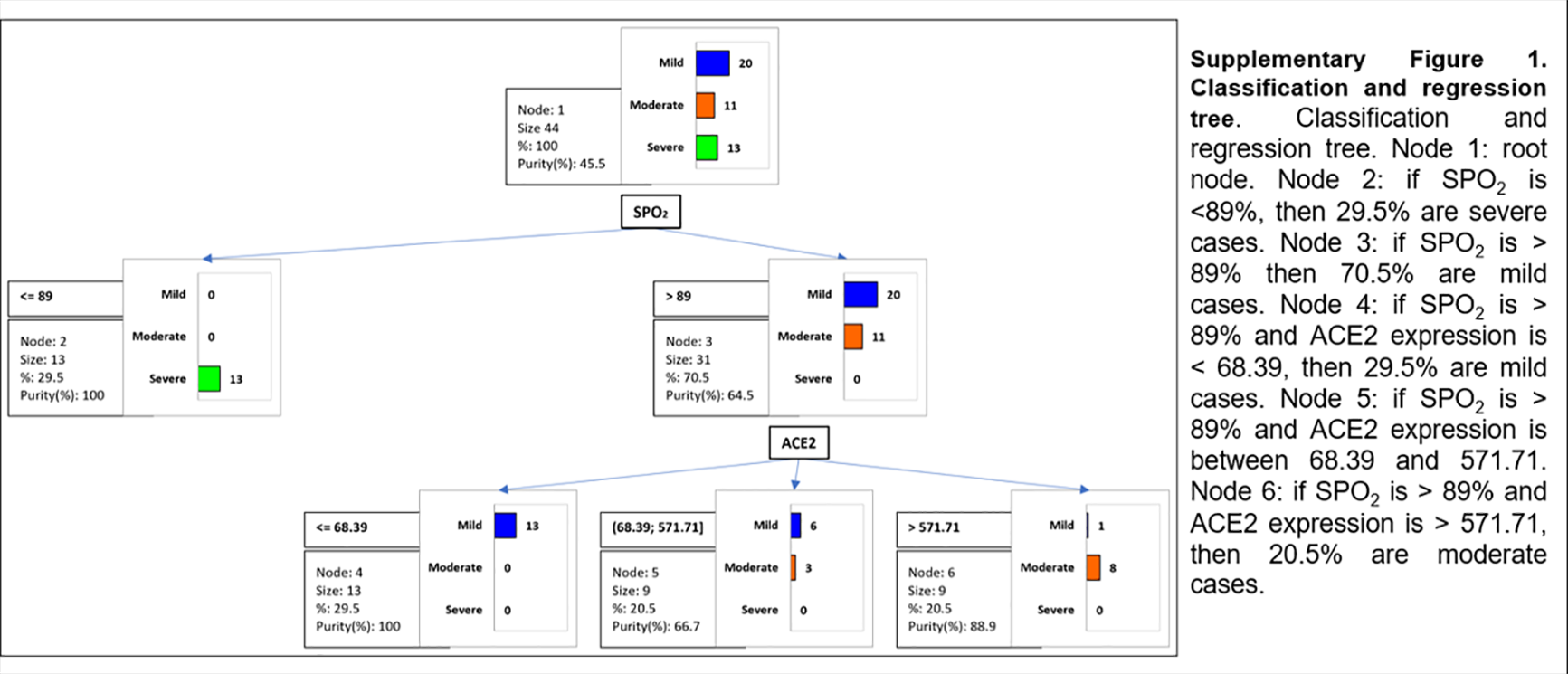

Supplement: Supplementary file 1 — Additional file 1: Supplementary Figure 1. [file 12879_2023_8573_MOESM1_ESM.tif]
